# A prospective, self-controlled study of sub-plateau heart rate variability in healthy adults

**DOI:** 10.3389/fphys.2024.1464144

**Published:** 2024-12-09

**Authors:** Xianglin Ye, Hao Liu, Haixia Yang, Hongyang Zhang, Meiting Gong, Zhen Duan, Yan Fu, Shiqiang Xiong, Xiaoping Dan, Haifeng Pei

**Affiliations:** ^1^ Department of Cardiology, The General Hospital of Western Theater Command, Chengdu, China; ^2^ Department of Clinical Medicine, Southwest Medical University, Luzhou, China; ^3^ Department of Pediatrics, The General Hospital of Western Theater Command, Chengdu, China; ^4^ Patients Management Department, The General Hospital of Western Theater Command, Chengdu, China; ^5^ Department of Cardiology, Cardiovascular Disease Research Institute of Chengdu, Chengdu Third People’s Hospital/Affiliated Hospital of Southwest Jiao Tong University, Chengdu, China; ^6^ Si Chuan International Travel Health Center (Port Clinic of Cheng Du Customs), Chengdu, China

**Keywords:** sub-plateau, acclimatization/de-acclimatization, heart rate variability, sympathetic/parasympathetic nerves, circadian variations

## Abstract

**Background:**

The low-pressure, hypoxic environment characteristic of high-altitude regions significantly affects the cardiovascular and autonomic nervous system functions of individuals, consequently impairing their sleep quality. Heart rate variability, a non-invasive indicator of autonomic nervous system activity and balance within the cardiovascular system, has not been thoroughly investigated in terms of its patterns during acclimatization and de-acclimatization phases for individuals traveling to and residing in high-altitude areas and its relationship with sleep stability.

**Methods:**

Data was collected from 22 medical staff members who traveled from Chengdu to Yecheng, with measurements taken before leaving Chengdu, 1 week in Yecheng, 3 months in Yecheng, and 1 week after returning to Chengdu. The study analyzed changes in heart rate variability during acclimatization and de-acclimatization at 1,400 m above sea level. It also examined arrhythmia and sleep disorders based on circadian groups.

**Results:**

1. Following 1 week of acclimatization to the sub-plateau environment of Yecheng, significant decreases were observed in SDANN, SDNN and SD2 indices compared to departure from Chengdu (*P* < 0.05). After 3 months of sub-plateau acclimatization, these indices significantly increased (*P* < 0.05). Upon returning to Chengdu and undergoing de-acclimatization for 1 week, these indices further significantly increased (*P* < 0.05). 2. During the period of sub-plateau acclimatization and de-acclimatization, significant changes were noted in average heart rate and minimum heart rate (*P* < 0.05), with the average heart rate showing a continuous decrease and the minimum heart rate exhibiting an initial increase followed by a decrease. No significant changes were observed in maximum heart rate or the incidence of arrhythmias (*P* > 0.05). 3. When stratified by day and night, the trends for SDANN, RMSSD, and TP were consistent with the overall trend at night (*P* < 0.05), but opposite during the day (*P* < 0.05). 4. During the sub-plateau acclimatization period, stable sleep duration was significantly reduced, and increased markedly after de-acclimatization, although it did not return to pre-acclimatization levels (*P* < 0.05).

**Conclusion:**

Acclimatization to the sub-plateau environment of Yecheng affects the autonomic nervous system, heart rate, and sleep in healthy adults. De-acclimatization can ameliorate these effects. Furthermore, the impact of sub-plateau acclimatization on the autonomic nervous system exhibits a distinct circadian rhythmicity.

## 1 Introduction

Chinese plateau terrain encompasses nearly half of the country’s total land area. As a result of economic development and improvements in transportation infrastructure in these highland regions, The number of individuals has seen a notable rise from lowland areas who are now traveling to and residing in these high-altitude regions. Presently, there is a substantial population of outsiders residing and employed in regions located at altitudes exceeding 4,000 m above sea level, thereby facing the health risks associated with elevated altitudes. Previous research studies have demonstrated that the low-pressure, low-oxygen environment at high altitudes has an impact on the human cardiovascular system and the functioning of the autonomic nervous system (Hainsworth and Drinkhill, 2007), thereby affecting sleep quality (Kong et al., 2018). Conversely, studies have indicated that training at sub-plateau levels can enhance the physical capabilities and performance of athletes (Ma et al., 2023). Therefore, it is now a pressing task to explore the precise impacts of the plateau environment on the human body. Heart rate variability (HRV) serves as a non-invasive measure to evaluate the activity and balance of the autonomic nervous system within the cardiovascular system (Shaffer et al., 2014; Kim et al., 2018). It reflects the sinus node autonomy, which is regulated by the autonomic nervous system, by measuring variations in interbeat intervals. Recent advancements in sensor technology have greatly facilitated the collection of human physiological data, leading to a surge in research on HRV. However, it is unclear that the pattern of HRV and its association with sleep stability in individuals who travel to and reside in sub-plateau regions during acclimatization and de-acclimatization. Therefore, the objective of this research was to examine the evolving patterns and features of electrocardiographic and sleep parameters in a cohort of well individuals who moved from Chengdu, Sichuan Province to Yecheng, Xinjiang .

**FIGURE 1:**
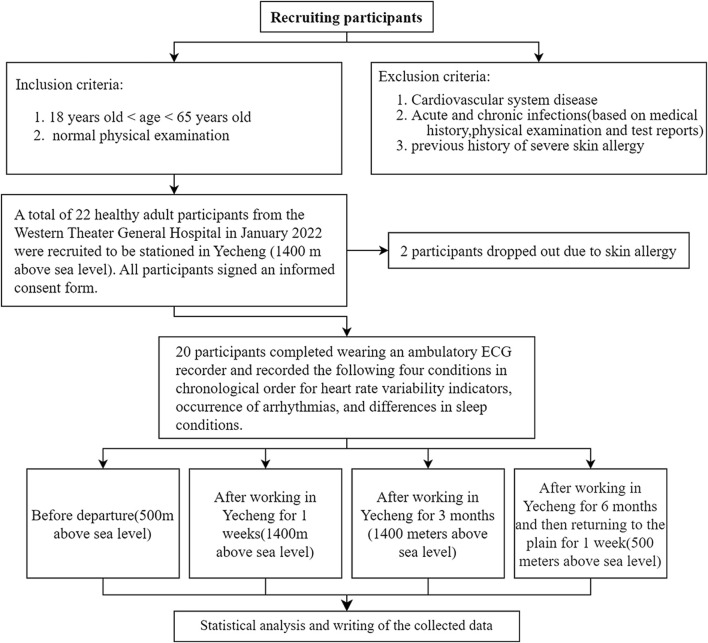
Experimental flow chart.

## 2 Materials and methods

### 2.1 Experiment design

Enrolled participants were provided with ambulatory electrocardiogram (ECG) recorders to wear four times by professionally trained technicians: before departing from Chengdu (500 m above sea level), after 1 week of stay in Yecheng (1,400 m above sea level), after 3 months of stay in Yecheng (1,400 m above sea level), and after returning to Chengdu for 1 week following 6 months of stay in Yecheng. Yecheng recorded an average atmospheric pressure of 86.59 kPa (ranging from 86.3 to 86.9 kPa), an average relative humidity of 37.3% (varying between 28% and 61%), and an average temperature of 16°C (spanning from −7°C to 32°C) during the period of observation. Each time, the recorder was worn from 8:00 a.m. to 8:00 a.m. the next day. Based on previous research (Liu et al., 2018), we defined 1 week after staying in Yecheng and 3 months after staying in Yecheng as Initial Acclimation and Basic Acclimation, respectively. The period of 1 week after returning to the flatland was defined as De-acclimation. The changes in HRV, sleep patterns, heart rate, and arrhythmia indexes were examined before and after each of the four experimental periods for the participants. Furthermore, data on ambulatory ECG during the day (8:00 a.m. to 8:00 p.m.) and night (8:00 p.m. to 8:00 a.m.) were gathered for the purpose of examining variations in heart rate variability throughout the acclimation and de-acclimation periods.

### 2.2 Research object and experimental flow chart

This is a prospective cohort study that recruited 22 healthy adult medical staff members who relocated from a hospital in Chengdu, Sichuan Province (500 m above sea level) to a hospital in Yecheng, Xinjiang (1,400 m above sea level) for support between January 2022 and August 2022. Accroding to the inclusion and exclusion criteria from Figure 1, 20 participants successfully completed the trial. The cohort details are follows: 12 males and 8 females, ages ranging from 29 to 48 years old (mean age: 36.60 ± 4.90 years), and BMI ranging from 18.26 to 26.64 kg/m^2^ (mean BMI: 21.93 ± 2.32 kg/m^2^). The study adheres to the principles outlined in *the Declaration of Helsinki on Human Experiments* and received approval from the Ethics Committee of the General Hospital of Western Theater (registration no: 2022EC2-Ky051).

### 2.3 ECG signals and sleep detection

The participants’ 24-hour ECG signals and sleep patterns were continuously monitored using a smartphone application called Guanxin, developed by Chengdu XinhuiJuyuan Technology Co., Ltd. The data was analyzed used an automatic algorithm to correct artifacts in the R-R interval sequence, as well as identify and fix ectopic and misaligned beats in normal sinus rhythm. This was accomplished by interpolating the R-R values without replacing more than 5% of the total heartbeats in the R-R period. HRV time-domain indexes as follows: SDNN: standard diviation of NN intervals, Standard deviation of all 24 h sinus RR intervals, negatively correlated with sympathetic tension. SDANN: five-minute R-R interval means, Standard deviation of the mean of sinus RR intervals per 5-min segment over 24 h, negatively correlated with sympathetic tension. RMSSD: root mean square successive difference, Root mean square of all sinus RR interval differentials in 24 h, positively correlated with vagal tension (Shaffer and Ginsberg, 2017). PNN50: percentage of adjacent NN intervals differing by more than 50 ms, Percentage of the number of two adjacent normal sinus RR intervals with a differential >50 ms in 24 h. The computerized automatic detection and calculation of sleep metrics encompass sleep efficiency, sleep quality, rapid eye movement period, stable sleep duration, and unstable sleep duration. Frequency domain metrics were calculated using the Fast Fourier Transform. TP represents the total power, LF represents the low frequency power that is positively correlated with vagal tension and pressure-sensitive reflex activity (Goldstein et al., 2011; Reyes del Paso et al., 2013). HF represents the high frequency power, HFnorm represents the standardized high frequency power, LFnorm represents the standardized low frequency power, and VLF represents the very low frequency power. Additionally, the nonlinear metrics by analyzing detrends fluctuations such as SD1, SD2, approximate entropy, and sample entropy. The measurements and analysis were performed by two professionally trained operators.

### 2.4 Statistical methods

SPSS software (SPSS 26.0, SPSS Inc.) was used for the statistical analysis. The initial normality and homogeneity of variance tests are performed. Data following a normal distribution were presented as mean ± standard deviation and compared using repeated-measures ANOVA. Non-parametric rank sum tests are used for data that do not conform to a normal distribution. Numerical data were presented as percentages and compared using the chi-square test. A two-factor repeated-measures ANOVA was utilized to compare the cyclic patterns in time domain, frequency domain, and nonlinear indexes at varying altitudes, with factors including day, night, and altitude. Statistical significance was defined as *P* < 0.05.

## 3 Results

### 3.1 Analysis of heart rate variability indexes during sub-plateau acclimatization and de-acclimatization

The results from Table 1 indicated that SDNN, SDANN, RMSSD, SD1, and SD2 were significantly lower (*P* < 0.05) after 1 week in Yecheng compared to the measurements taken before leaving Chengdu. The analysis showed an increase in sympathetic tension and a decrease in vagal tension after 1 week of acclimatization at the 1,400 m sub-plateau, characterized by sympathetic excitation. Conversely, after 3 months of acclimatization in Yecheng, the SDNN, SDANN and SD2 significantly increased and surpassed the values measured before leaving Chengdu (*P* < 0.05), suggesting an elevation in vagal tone and a reduction in sympathetic tone. This indicated that vagal excitation was dominant during this period. Upon returning to Chengdu for 1 week, the SDNN, SDANN and SD2 significantly increased again (*P* < 0.05) compared to the measurements taken after 3 months in Yecheng, and there was alsoa tendency of increase in RMSSD and SD1, indicating further enhancement of vagal tension and further decrease in sympathetic tension during de-acclimatization. The LF, which is an effective measure of carotid sinus tension, increased with time during the stay in Yecheng (*P* < 0.05) until it decreased after 1 week of returning to Chengdu (*P* < 0.05), although it did not return to the level before leaving Chengdu. In contrast, the total autonomic regulatory capacity, represented by TP, showed a tendency to decrease and then increase. Even 1 week after returning to Chengdu, TP continued to increase. These findings suggested that sub-plateau acclimatization in healthy adults dynamically regulated sympathetic/parasympathetic excitability and carotid sinus tension, while de-acclimatization reversed this regulation.

**TABLE 1:** Comparison of time-domain and frequency-domain indexes between acclimatization and de-acclimatization.

Variable	Before leaving chengdu	After 1 week in yecheng	After 3 months in yecheng	After 1 week back to chengdu	*F* -value	*P*-value
SDNN(ms)	156.67 ± 40.98	141.91 ± 45.53	172.27 ± 54.53	191.09 ± 52.72	3.604	0.021*
SDANN(ms)	147.69 ± 47.80	125.5 ± 45.6	158.02 ± 54.24	181.12 ± 58.82	3.871	0.016*
RMSSD (ms)	32.61 ± 13.42	32.18 ± 16.33s	37.7 ± 18.1	41.97 ± 15.57	1.404	0.255
LF (ms2)	766.57 ± 357.58	812.90 ± 494.49	1,049.31 ± 549.96	1,029.88 ± 406.35	3.598	0.021*
SD1	23.07 ± 9.50	22.76 ± 11.55	26.65 ± 12.81	29.71 ± 11.02	1.408	0.254
SD2	220.21 ± 57.72	199.31 ± 63.57	242.09 ± 76.25	268.52 ± 74.1	3.635	0.020*
PNN50(%)	11.95 ± 10.30	11.97 ± 11.62	14.44 ± 12.28	17.69 ± 10.63	0.838	0.481
HF(ms^2^)	454.51 ± 386.60	445.84 ± 447.46	549.51 ± 534.43	628.17 ± 499.54	0.487	0.693
TP (ms^2^)	15,216.15 ± 7,073.63	13,486.56 ± 6,096.93	16,955.11 ± 7,850.06	18,439.79 ± 8,552.75	1.019	0.394
VLF (ms^2^)	13,942.66 ± 6,787.44	12,186.42 ± 5,522.98	15,304.21 ± 7,470.58	16,724.23 ± 8,332.37	0.876	0.461
LFnorm (ms^2^)	63.41 ± 13.39	66.64 ± 12.05	67.09 ± 11.75	63.1 ± 11.53	0.813	0.494
HFnorm (ms^2^)	32.47 ± 12.64	29.49 ± 12.31	29.35 ± 11.81	33.17 ± 11.55	0.622	0.605
approximate entropy	0.66 ± 0.22	0.71 ± 0.17	0.67 ± 0.11	0.67 ± 0.18	5.576	0.003
sample entropy	0.50 ± 0.20	0.56 ± 0.17	0.49 ± 0.1	0.47 ± 0.15	0.713	0.550

Note: SDNN: the standard deviation of the normal sinus RR, interval over a 24-hour period, is negatively correlated with sympathetic tension. SDANN: the standard deviation of the mean of normal sinus RR, intervals per 5-minute segment over a 24-hour period, is negatively correlated with sympathetic tension. RMSSD: the square root of the mean of the sum of squares of normal sinus RR, interval differences over 24 h, is positively correlated with vagal tension (Shaffer and Ginsberg, 2017). LF: which represents low-frequency power ranging from 0.04 to 0.15 Hz, is positively correlated with vagal tension and pressure-sensitive reflex activity (Goldstein et al., 2011; Reyes del Paso et al., 2013). SD1 and SD2: nonlinearly analyzed indexes that are positively correlated with the degree of vagus nerve activity. PNN50: represents the percentage of the total number of RR, intervals in a 24-hour period with a difference between two adjacent RR, intervals greater than 50 m. HF: which represents high-frequency power ranging from 0.15 to 0.40 Hz, is positively correlated with vagal tension. TP: or Total Power, reflects the variance of all RR, intervals and reflects the degree of overall heart rate variability within the frequency range of ≤0.40 Hz. VLF: very low frequency power, is the term used to describe the power within the frequency range of 0.003 Hz–0.04 Hz. LFnorm: the standardized low-frequency power, = HF/(total power-VLF)×100. HFnorm: the standardized high-frequency power, = HF/(total power-VLF)×100.

*P < 0.05.

** *P* < 0.01; n = 20.

### 3.2 Analysis of heart rate and arrhythmia during the plateau acclimatization and de-acclimatization process

According to Table 2, the mean heart rate (MeanHR) of the participants consistently decreased over time (*P* < 0.05) during their stay in Yecheng for sub-plateau acclimatization, as well as during the de-acclimatization process back to Chengdu. On the other hand, the minimum heart rate (MinHR) increased after 1 week of stay in Yecheng and then consistently decreased over time (*P* < 0.05). However, the occurrence of arrhythmias, including maximum heart rate, supraventricular premature contractions, ventricular premature contractions, supraventricular and ventricular tachycardias, did not show any statistical significance (*P* > 0.05) across all time periods. These findings suggested that the 1,400 m sub-plateau environment might enhance the circulatory reserve capacity in healthy adults without increasing the risk of heart disease.

**TABLE 2:** Comparison of heart rate and arrhythmias between sub-plateau acclimatization and de-acclimatization.

Variable	Before leaving Chengdu	After 1 week in Yecheng	After 3 months in Yecheng	After 1 week back to Chengdu	*F*-value /*Q*-value	*P*-value
MeanHR	76.30 ± 7.98	74.25 ± 9.62	72.35 ± 9.31	68.27 ± 6.52	8.577	<0.001**
MinHR	52.60 ± 7.96	54.15 ± 7.80	49.24 ± 6.44	46.87 ± 6.21	8.120	<0.001**
MaxHR	134 ± 17.48	128.30 ± 19.07	132.18 ± 15.77	116.93 ± 31.79	1.304	0.288
SVPCs (n)	44.2 ± 90.52	118.35 ± 426.07	36.29 ± 36.38	17.87 ± 18.54	1.057	0.380
^a^VPCs (cases)	50%(10)	40%(8)	53%(9)	40%(6)	0.853	0.374
^a^VT(cases)	5%(1)	0	0	0	1.000	0.404
^a^SVT (cases)	25%(5)	25%(5)	29.4%(5)	13.3%(2)	2.090	0.119

Note: MeanHR: Mean heart rate; MinHR: Minimum heart rate; MaxHR: Maximum heart rate; SVPCs: supraventricular premature contractions; VPCs: ventricular premature contractions; VT: Ventricular tachycardia; SVT: Supraventricular tachycardia.

^a^ A percentage represents the number of individuals with each type of arrhythmia as a percentage of the total number of individuals.

*P < 0.05.

**P < 0.01; n = 20.

### 3.3 Characterization of circadian heart rate variability in plateau acclimatization and de-acclimatization

A repeated measures ANOVA was conducted to examine the primary impact of circadian group, the main effect of tests number, and the interaction between circadian group and number of tests for each metric, with measurement time point as a within-subjects variable and circadian group as a between-subjects factor. The results from Table 3 indicated that the main effect of number of tests was significant (*P* < 0.05) for participants’ SDNN, SDANN, RMSSD, TP, SD1, and SD2. Additionally, SDNN and SD2 showed a significant interaction between circadian group and number of tests (*P* < 0.05), while LF did not in circadian group. These findings suggested that sympathetic tension tended to decrease gradually during the daytime, followed by an increase and then a decrease at night for participants residing in Yecheng. On the other hand, vagal tension tended to increase gradually during the daytime, followed by a decrease and then an increase at night for participants residing in Yecheng. After returning to Chengdu for 1 week, sympathetic nerve tension decreased and vagal nerve tension increased compared to the levels before leaving Chengdu. Furthermore, the total autonomic nerve regulation, represented by TP, tended to gradually increase during the daytime with residence time, followed by a decrease and then an increase at night. This pattern indicated a decrease during the daytime but still higher than the pre-acclimatization levels, and an increase at night after returning to Chengdu for 1 week. Carotid sinus tension, as represented by LF, gradually increased throughout the acclimatization and de-acclimatization process, regardless of day and night, and then decreased after returning to Chengdu for 1 week, although it remained higher than the levels before leaving Chengdu. While SDANN, RMSSD, and LF showed a significant effect of test time point (*P*< 0.05), their interaction effect with group by test time point was not significant (*P* > 0.05). These results suggested that the trend of sympathetic/parasympathetic tension during 1,400 m sub-plateau acclimation and de-acclimation in the healthy adult population at night was consistent with the overall trend, whereas the trend during the daytime was opposite to the overall trend. Additionally, no circadian difference in carotid sinus tension was observed.

**TABLE 3:** Comparison of daytime and nighttime time-domain indexes during acclimatization and de-acclimatization.

Variable		Before leaving Chengdu	After 1 week in Yecheng	After 3 months in Yecheng	After 1 week back to Chengdu	*F*-value	*P*-value	bias square
SDNN(ms)	Day	109.71 ± 25.24	117.79 ± 30.51	144.15 ± 41.87	125.05 ± 31.04	—		
	Night	155.01 ± 56.09	132.04 ± 48.83	169.26 ± 49.06	190.31 ± 66.62			
	Group Main Effect	—				12.915	<0.001**	0.316
	Number of tests main effect					7.007	<0.001**	0.200
	Group*Number of tests					2.785	0.046*	0.090
SDANN(ms)	Day	93.80 ± 31.30	101.54 ± 28.73	129.12 ± 41.79	108.27 ± 31.82	—		
	Night	131.88 ± 52.17	108.86 ± 44.35	150.73 ± 46.19	161.78 ± 61.73			
	Group Main Effect	—				10.258	0.003*	0.268
	Number of tests main effect					5.833	<0.001**	0.172
	Group*Number of tests					1.955	0.127	0.065
RMSSD(ms)	Day	27.74 ± 11.97	28.60 ± 14.40	33.03 ± 13.81	32.54 ± 11.59	—		
	Night	38.31 ± 16.79	35.70 ± 19.17	41.57 ± 23.19	50.79 ± 20.52			
	Group Main Effect	—				8.680	0.006*	0.237
	Number of tests main effect					3.347	0.023*	0.107
	Group*Number of tests					1.087	0.359	0.037
LF(ms2)	Day	728.62 ± 331.65	754.15 ± 455.49	940.29 ± 420.19	834.67 ± 293.58	—		
	Night	807.13 ± 441.47	867.91 ± 553.56	1154.41 ± 729.79	1219.09 ± 551.96			
	Group Main Effect	—				3.291	0.080	0.105
	Number of tests main effect					11.310	<0.001**	0.288
	Group*Number of tests					1.674	0.179	0.056
HF(ms2)	Day	291.83 ± 308.69	362.56 ± 423.38	426.88 ± 387.77	338.06 ± 293.34	—		
	Night	636.09 ± 521.93	525.28 ± 503.97	655.71 ± 689.20	910.18 ± 745.30			
	Group Main Effect	—				7.690	0.010	0.215
	Number of tests main effect					1.077	0.308	0.037
	Group*Number of tests					1.339	0.257	0.046
TP(ms2)	Day	9213.68 ± 5193.37	10297.55 ± 4574.16	13589.28 ± 6331.88	11922.67 ± 6001.99	—		
	Night	13009.70 ± 7062.46	10402.23 ± 6756.16	14625.44 ± 8503.98	16010.69 ± 7506.04			
	Group Main Effect	—				2.585	0.119	0.085
	Number of tests main effect					2.883	0.041	0.093
	Group*Number of tests					0.389	0.761	0.014
SD1	Day	19.61 ± 8.48	20.22 ± 10.19	23.37 ± 9.77	23.01 ± 8.20	—		
	Night	27.10 ± 11.88	25.24 ± 13.56	29.41 ± 16.42	35.91 ± 14.5			
	Group Main Effect	—				8.675	0.006	0.237
	Number of tests main effect					3.342	0.023	0.107
	Group*Number of tests					1.087	0.359	0.037
SD2	Day	153.72 ± 35.71	165.22 ± 42.42	202.46 ± 58.60	175.22 ± 43.69	—		
	Night	217.38 ± 78.88	184.92 ± 68.03	237.42 ± 68.02	266.59 ± 93.52			
	Group Main Effect	—				12.226	0.002	0.304
	Number of tests main effect					5.786	0.001	0.171
	Group*Number of tests					3.097	0.031	0.100
	Day	0.82 ± 0.28	0.75 ± 0.20	0.71 ± 0.13	0.82 ± 0.21	—		
	Night	0.78 ± 0.22	0.85 ± 0.23	0.74 ± 0.19	0.83 ± 0.19			
approximate entropy	Group Main Effect	—				0.538	0.469	0.019
	Number of tests main effect					1.817	0.163	0.061
	Group*Number of tests					0.375	0.728	0.013
sample entropy	Day	0.65 ± 0.25	0.58 ± 0.18	0.52 ± 0.10	0.62 ± 0.16	—		
	Night	0.66 ± 0.22	0.72 ± 0.24	0.57 ± 0.20	0.69 ± 0.19			
	Group Main Effect	—				3.397	0.076	0.108
	Number of tests main effect					2.729	0.063	0.089
	Group*Number of tests					0.413	0.700	0.015

Note: SDNN, the standard deviation of the normal sinus RR interval over a 24-hour period, is negatively correlated with sympathetic tension; SDANN, the standard deviation of the mean of normal sinus RR intervals per 5-minute segment over a 24-hour period, is negatively correlated with sympathetic tension; RMSSD, the square root of the mean of the sum of squares of normal sinus RR interval differences over 24 hours, is positively correlated with vagal tension; LF, which represents low-frequency power ranging from 0.04 to 0.15 Hz, is positively correlated with vagal tension and pressure-sensitive reflex activity. HF: which represents high-frequency power ranging from 0.15 to 0.40 Hz, is positively correlated with vagal tension; TP, or Total Power, reflects the variance of all RR intervals and reflects the degree of overall heart rate variability within the frequency range of ≤0.40 Hz. SD1 and SD2: nonlinearly analyzed indexes that are positively correlated with the degree of vagus nerve activity.

*P < 0.05.

** *P* <0.01; n = 20.

### 3.4 Analysis of the impact of sub-plateau acclimatization and de-acclimatization on sleep

Table 4 presents that the percentage of stable sleep period, sleep efficiency, and sleep quality gradually declined in YeCheng. However, after returning to Chengdu for 1 week, these measures gradually improved. Notably, the percentage of stable sleep period exhibited the most significant change (*P*< 0.05), indicating a gradual decrease during the 1,400 m sub-plateau acclimatization and subsequent recovery after de-acclimatization. Conversely, the proportion of rapid eye movement (REM) period, unstable sleep, and the apnea-hypopnea index (AHI) did not demonstrate any statistical significance (*P* > 0.05), despite increasing in YeCheng and gradually decreasing 1 week after returning to Chengdu. These findings suggested that 1,400 m sub-plateau acclimatization and de-acclimatization has an impact on the occurrence of sleep disorders in healthy adults, with the proportion of stable sleep phase being the most significantly affected.

**TABLE 4:** Comparison of sleep during sub-plateau acclimatization and de-acclimatization

Variable	Before leaving Chengdu	After 1 week in Yecheng	After 3 months in Yecheng	After 1 week back to Chengdu	*F*-value	*P*-value
^a^Percentage of stable sleep period (%)	50.40 ± 15.38	43.63 ± 19.01	35.74 ± 16.22	39.56 ± 19.81	3.929	0.015*
^a^Rapid eye movement period percentage (%)	8.70 ± 5.09	11.63 ± 8.42	12.22 ± 7.10	12.46 ± 12.06	2.079	0.171
^a^Percentage of unstable sleep (%)	11.04 ± 5.78	11.83 ± 8.89	15.55 ± 10.99	10.55 ± 7.69	1.055	0.379
sleep efficiency (%)	70.12 ± 15.97	67.07 ± 21.09	63.21 ± 16.94	62.17 ± 23.52	0.275	0.843
Times of awakenings (n)	15.15 ± 8.44	14.58 ± 9.83	16.65 ± 7.69	15.38 ± 9.14	0.053	0.984
AHI	11.34 ± 7.38	23.54 ± 36.50	32.48 ± 45.51	16.01 ± 6.83	3.792	0.072
Quality of sleep	79.70 ± 10.53	75.74 ± 14.09	73.65 ± 10.53	72.81 ± 15.46	0.733	0.538

Note: AHI, apnea-hypopnea index, average number of occurrences of apnea plus hypopnea for each hour of sleep.

^a^ Percentage refers to the corresponding time as a percentage of total sleep duration.

* *P* < 0.05.

** *P* < 0.01; n = 20.

## 4 Discussion

Heart rate variability (HRV) serves not only as a non-invasive metric for assessing the activity and balance of the autonomic nervous system within the cardiovascular system, but also possesses the functionality to predict prognoses of diseases including acute myocardial infarction. Beyond this, HRV has significant applications in the realms of psychological research, sports rehabilitation, and healthy aging (Laborde et al., 2022). Concurrently, advancements in sensor and cloud computing technologies have rendered it feasible to study the patterns of HRV changes among populations across different regions and altitudes. In the normoxic environment of the flatland, the sympathetic and parasympathetic excitability of the body are relatively balanced. Nevertheless, when exposed to the hypoxic conditions of the plateau, the autonomic nervous system experiences adaptive changes, and any lack of balance can result in dysfunction across different organ systems (Todd, 1991; Naeije, 2010). Plateau stress increases sympathetic tension, decreases vagal tension, and reduces sensitivity to neurohumoral regulation, resulting in greater decreases in heart rate variability and even severe arrhythmias (Goldstein et al., 2011; Schmid et al., 2015; Woods et al., 2011). In this study, we observed that when participants worked in a sub-plateau environment for 1 week, sympathetic tension was enhanced and vagal tension was weakened, indicating a predominance of sympathetic stress. After acute entry into the plateau, the body was in a state of emergency and more susceptible to fatigue due to increased sympathetic excitability caused by low-pressure hypoxia and cold. Consistent with this Chen et al. (2020), analyzed ECG data from 30 miners in China who were working at an elevation of 3,500–4,000 m and found that sympathetic tension increased while parasympathetic tension decreased during fatigue among plateau miners. Additionally, low-pressure hypoxia could enhance pulmonary vascular remodeling, leading to pulmonary hypertension (Grimminger et al., 2017). Therefore, it is important to take precautions to keep warm and rest after ascending to the plateau, and highland workers with cardiorespiratory diseases should avoid rapid increases in altitude. Three months of working at a sub-plateau resulted in increased vagal tension and decreased sympathetic tension in participants, indicating a predominance of vagal nerve stress, similar to the findings of Boushel et al. (2001), which showed that exposure to 5,000 m altitude for 9 weeks resulted in increased parasympathetic activity and reduced heart rate during plateau acclimation. After participants had worked on the sub-plateau for 6 months and returned to the Chengdu flatland for 1 week, their vagal nerve tension further increased and sympathetic nerve tension further decreased compared to that after 3 months of sub-plateau work. Consequently, it is evident that appropriate flatland recuperation in highland dwelling groups is a highly effective therapeutic measure for restoring sympathetic and parasympathetic balance. In our study, we also observed that the low-frequency power metric (LF) increased upon reaching the plateau and only slightly decreased upon returning to the flatlands, with no significant circadian differences. Goldstein et al. (2011) demonstrated that LF responded to the level of carotid sinus pressure receptor reflexes. When blood pressure rose in humans, pressure receptors on the carotid sinus were stimulated, leading to an elevation in vagal tone and a reduction in sympathetic tone. The LF was found to increase in individuals with normal pressure reflexes following carotid sinus stimulation from neck pumping, whereas no elevation of LF was observed in those with impaired pressure reflexes (Sleight et al., 1995). Previous research suggested that increased altitude could cause a rise in blood pressure. Bilo et al. (2019) demonstrated that exposure of humans to approximately 2,000 m above sea level may be a notable rise in blood pressure within a 24-hour timeframe. These findings suggested that the carotid sinus pressure reflex of the participants was significantly influenced by the plateau environment, and LF might be inherently correlated with plateau blood pressure.

Dynamic changes in sympathetic and parasympathetic tension over time following rapid ascent to high altitudes can have a significant impact on heart rate. The present study demonstrated that during sub plateau acclimatization and subsequent return to lower altitudes for de-acclimatization, the mean heart rate of participants gradually decreased over time, while the minimum heart rate initially increased after the rapid ascent and then gradually declined. Siebenmann et al. (2017) identified that the main reason for the rise in heart rate after 15–18 days at an altitude of 3,454 m was a reduction in parasympathetic activity in the heart Sander, (2016). observed an elevation in sympathetic activity of the myocardium with a concurrent rise in heart rate in eight Danish lowlanders after 4 weeks of exposure to an altitude exceeding 5,000 m Li et al. (2021). suggested that the regulation of the cardiovascular autonomic nervous system decreased over time during short-term hypoxic exposures, with the number and strength of sympathetic and parasympathetic modulations tending to equalize. The disparity in results could potentially be attributed to the lower altitude and younger age of the participants involved in this study. While hypoxia and sympathetic excitation led to an elevation in minimum heart rate during the initial week, the average heart rate of the participants actually decreased due to reduced activity following the rapid ascent to the sub-plateau. Additionally, the participants consisted of healthy adults who exhibited tolerance to hypoxia, resulting in a gradual decline in their heart rate as parasympathetic tension increased over the acclimatization. Furthermore, there was no statistical difference in the related indications of arrhythmia. These findings suggested that the circulatory reserve capacity of healthy adults increases at approximately 1,400 m altitude without an elevated risk of cardiac disease. This partially supports the notion that athletes who choose to train at this altitude are more likely to enhance their training endurance and improve performance. Similarly, the findings of Ma et al. (2023) also supported this point that the physical functioning and athletic performance of male ski athletes showed significant improvement, after 6 weeks of training at lower altitudes.

Normal sleep is characterized by cycles of non-rapid eye movement (NREM) and rapid eye movement (REM) sleep, which are heavily linked to fluctuations in heart rate. During NREM sleep, the body is primarily regulated by the parasympathetic system, resulting in a relatively stable cardiovascular system. In contrast, during REM sleep, there is a rise in sympathetic activity and a reduction in cardiovascular stability (Cabiddu et al., 2012). Therefore, sleep quality can serve as an accurate indicator of the functional state of the sympathetic and vagal nervous systems, and *vice versa*. Our study revealed that sub-plateau acclimatization significantly reduced the proportion of stable sleep periods in participants, and this reduction persisted even after de-acclimatization. Furthermore, we observed that upon entering the sub-plateau, sympathetic tension gradually decreased during the daytime but increased and then decreased at night, while vagal tension gradually increased during the daytime but decreased and then increased at night. Upon returning to the flatland, sympathetic tension decreased and vagal tension increased compared to pre-acclimatization levels, but still followed the circadian pattern of higher sympathetic tension during the day and stronger vagal tension at night. Additionally, we found that during our stay in Yecheng, the total autonomic regulatory capacity, represented by TP, tended to increase gradually during the day with the duration of stay, but decreased and then increased at night. After returning to Chengdu for 1 week, TP decreased during the daytime but remained higher than pre-acclimatization levels, while continuing to increase at night. Consistent with our findings, Mantzios et al. (2023) demonstrated that a 2-week descent from 7,000 m to 4,000 m resulted in a significant increase in nighttime TP, whereas the following 5-day ascent from 4,000 m to 7,000 m led to a significant decrease in nighttime TP. Jafarian et al. (2008) reported that an altitude of 3,500 m caused frequent nocturnal awakenings and sleep disturbances in 46% of the observed population. Another study indicated that 32% of individuals experienced insomnia during their initial visit to an altitude of 3,700 m from 500 m (Bloch et al., 2015). Bilo et al. (2015) demonstrated that at high altitudes, a further decrease in oxygen saturation during sleep triggered sympathetic activation to counteract the decrease in blood pressure, and psychological factors might also contribute to the development of these symptoms. As altitude increases, the decrease in oxygen partial pressure can cause hyperventilation to result in hypocapnia and respiratory alkalosis. And it caused a shift to the left of the oxygen dissociation curve, further reducing oxygen supply to cells and creating a cycle of “hypoxia-hyperventilation-hypoxia” (Burtscher et al., 2022a). Continuous positive-pressure ventilation can be employed to manage these sleep-related symptoms. Patients suffering from obstructive sleep apnea may experience improvements in the cycle of heart rate and blood pressure caused by hypoxia and hypercapnia through the use of continuous positive pressure ventilation and this treatment can also decrease sympathetic activity and increase parasympathetic activity (Dissanayake et al., 2022). Additionally, acetazolamide-induced stimulation of central chemoreceptors increases ventilation levels and arterial oxygen saturation at high altitudes, significantly reducing the occurrence and duration of apneic episodes (Hermand et al., 2021), thereby preventing acute and chronic mountain sickness. A meta-analysis demonstrated (Kong et al., 2018) that the non-benzodiazepine sedative-hypnotic drug zolpidem effectively improved sleep quality without affecting ventilation function and oxygen saturation, with a high level of safety. Therefore, it is recommended as a medication to improve sleep quality in individuals at acute and high altitudes. Animal experiments have shown (Hung et al., 2017) that exogenous melatonin supplementation reduced pulmonary arterial hypertension and inhibited the expression of pro-inflammatory factors. Scholars such as Calderon-Jofre et al. (2021) have demonstrated that increased altitude led to elevated melatonin levels in the population, which could serve as a marker of sleep quality. Burtscher et al. (2022b) have shown that melatonin could alleviate fatigue caused by hypoxia-induced sleep disorders. However, the lack of appropriate clinical trials necessitates further research to determine whether exogenous melatonin supplementation can improve sleep quality at high altitudes.

It is important to acknowledge the limitations of our study, including a small sample size, a limited age range of participants, and the absence of a sleep rating scale. The present study employs a single-subject design, which inherently limits its generalizability when employing multiple outcome measures. Consequently, it is imperative to acknowledge the constrained applicability of the findings within this context. Future research should aim to expand the sample size in order to establish a safe threshold for human plateau electrophysiology and offer health guidance for individuals traveling to plateaus.

In conclusion, our study demonstrated that acclimation to a sub-plateau altitude of 1,400 m had a dynamic effect on the sympathetic and parasympathetic nervous system (SPSNS), resulting in changes in heart rate and disruption of the stable sleep period in healthy adults. Conversely, de-acclimation to the sub-plateau altitude reversed the SPSNS regulation, leading to a decrease in heart rate and improvement in the stable sleep period. Additionally, the regulation of the SPSNS exhibited distinct diurnal and nocturnal variations. These findings provide a basis for investigating the electrophysiological characteristics of large-scale populations in sub-plateau environments.

## Data Availability

The data presented in the study are deposited in the Figshare repository as Pei, Haifeng (2024). HRV data. Figshare. Dataset. https://doi.org/10.6084/m9.figshare.27901710.v3.
